# Supplementary material for: Dissemination of carbapenemase-producing Enterobacterales in the community of Rawalpindi, Pakistan
Source: PLoS One. 2022 Jul 8;17(7):e0270707. doi: 10.1371/journal.pone.0270707 (PMC9269877; doi:10.1371/journal.pone.0270707)
Supplement: S1 Table — (DOCX) [file pone.0270707.s001.docx]

| **Antimicrobial classes** | **Antimicrobial Agents** | **CRE isolates (n=78)** | | | | | |
| --- | --- | --- | --- | --- | --- | --- | --- |
|  |  | **Sensitive** | | **Intermediate** | | **Resistant** | |
|  |  | **N** | **%** | **N** | **%** | **N** | **%** |
| β- lactams +  β-lactamase Inhibitors | Amoxicillin/clavulanic acid | 0 | 0 | 0 | 0 | 78 | 100 |
|  | Ticarcillin/clavulanic acid | 0 | 0 | 0 | 0 | 78 | 100 |
| Penicillins | Ticarcillin | 0 | 0 | 0 | 0 | 78 | 100 |
|  | Temocillin | 1 | 1 | 0 | 0 | 77 | 99 |
| Cephalosporins | Cefoxitin | 0 | 0 | 0 | 0 | 78 | 100 |
|  | Cefotaxime | 0 | 0 | 0 | 0 | 78 | 100 |
|  | Ceftazidime | 0 | 0 | 0 | 0 | 78 | 100 |
|  | Cefepime | 0 | 0 | 1 | 1 | 77 | 99 |
| Monobactams | Aztreonam | 9 | 12 | 7 | 9 | 62 | 79 |
| Carbapenems | Ertapenem | 0 | 0 | 0 | 0 | 78 | 100 |
|  | Imipenem | 5 | 6 | 1 | 1 | 72 | 92 |
| Aminoglycosides | Amikacin | 59 | 76 | 0 | 0 | 19 | 24 |
|  | Gentamicin | 54 | 69 | 0 | 0 | 24 | 31 |
| Quinolones | Nalidixic Acid | 1 | 1 | 0 | 0 | 77 | 99 |
|  | Ofloxacin | 1 | 1 | 1 | 1 | 76 | 97 |
| Sulfonamides | Trimethoprim/Sulphamethoxazole | 5 | 6 | 0 | 0 | 73 | 94 |
| Polymyxins | Colistin | 1 | 1 | 0 | 0 | 77 | 99 |

**S1 Table: Antimicrobial susceptibility profiling of CRE isolates**
