# Supplementary material for: Dissemination of carbapenemase-producing Enterobacterales in the community of Rawalpindi, Pakistan
Source: PLoS One. 2022 Jul 8;17(7):e0270707. doi: 10.1371/journal.pone.0270707 (PMC9269877; doi:10.1371/journal.pone.0270707)
Supplement: S2 Table — (DOCX) [file pone.0270707.s002.docx]

| **No. of Strains**  **S2 Table: Categorization of CRE isolates into XDR and MDR** | **%** | **Type of Resistance** | **In-vitro Phenotypic Resistance** | **In-vitro Phenotypic Susceptible** |
| --- | --- | --- | --- | --- |
| 47 | 60 | XDR | Penicillins+β-lactamase Inhibitors:  Amoxicillin/clavulanic acid,  Ticarcillin/clavulanic acid | Aminoglycosides: Amikacin,  Gentamicin |
|  |  |  | Penicillins: Ticarcillin, Témocillin | Polymyxins: Colistin |
|  |  |  | Cephalosporins: Cefoxitin, Cefotaxime, Ceftazidime, Cefepime |  |
|  |  |  | Monobactams: Aztreonam |  |
|  |  |  | Carbapenems: Ertapénem, Imipenem |  |
|  |  |  | Quinolones: Nalidixic acid, Ofloxacin |  |
|  |  |  | Sulfonamides: Trimethoprim/Sulphamethoxazole |  |
| 11 | 14 | XDR | Penicillins+β-lactamase Inhibitors:  Amoxicillin/clavulanic acid,  Ticarcillin/clavulanic acid | Polymyxins: Colistin |
|  |  |  |  |  |
|  |  |  | Penicillins: Ticarcillin, Témocillin |  |
|  |  |  | Cephalosporins: Cefoxitin, Cefotaxime, Ceftazidime, Cefepime |  |
|  |  |  | Aminoglycosides: Amikacin,  Gentamicin |  |
|  |  |  | Carbapenems: Ertapénem, Imipenem |  |
|  |  |  | Quinolones: Nalidixic acid, Ofloxacin |  |
|  |  |  | Monobactams: Aztreonam |  |
|  |  |  | Sulfonamides:  Trimethoprim/Sulphamethoxazole |  |
| 3 | 4 | XDR | Penicillins+β-lactamase Inhibitors:  Amoxicillin/clavulanic acid,  Ticarcillin/clavulanic acid | Monobactams: Aztreonam |
|  |  |  | Penicillins: Ticarcillin, Témocillin | Polymyxins: Colistin |
|  |  |  | Cephalosporins: Cefoxitin, Cefotaxime, Ceftazidime, Cefepime |  |
|  |  |  | Aminoglycosides: Amikacin,  Gentamicin |  |
|  |  |  | Carbapenems: Ertapénem, Imipenem |  |
|  |  |  | Quinolones: Nalidixic acid, Ofloxacin |  |
|  |  |  | Sulfonamides:  Trimethoprim/Sulphamethoxazole |  |
| 2 | 3 | XDR | Penicillins+β-lactamase Inhibitors:  Amoxicillin/clavulanic acid,  Ticarcillin/clavulanic acid | Sulfonamides: Trimethoprim/Sulphamethoxazole |
|  |  |  | Penicillins: Ticarcillin, Témocillin | Polymyxins: Colistin |
|  |  |  | Cephalosporins: Cefoxitin, Cefotaxime, Ceftazidime, Cefepime |  |
|  |  |  | Monobactams: Aztreonam |  |
|  |  |  | Carbapenems: Ertapénem, Imipenem |  |
|  |  |  | Aminoglycosides: Amikacin,  Gentamicin |  |
|  |  |  | Quinolones: Nalidixic acid, Ofloxacin |  |
| 1 | 1 | XDR | Penicillins+β-lactamase Inhibitors:  Amoxicillin/clavulanic acid,  Ticarcillin/clavulanic acid | Polymyxins: Colistin |
|  |  |  | Penicillins: Ticarcillin, Témocillin |  |
|  |  |  | Cephalosporins: Cefoxitin, Cefotaxime, Ceftazidime, Cefepime |  |
|  |  |  | Monobactams: Aztreonam |  |
|  |  |  | Carbapenems: Ertapénem, Imipenem |  |
|  |  |  | Aminoglycosides: Amikacin,  Gentamicin |  |
|  |  |  | Quinolones: Nalidixic acid, Ofloxacin |  |
|  |  |  | Sulfonamides:  Trimethoprim/Sulphamethoxazole |  |
| 5 | 6 | MDR | Penicillins+β-lactamase Inhibitors:  Amoxicillin/clavulanic acid,  Ticarcillin/clavulanic acid | Carbapenems: Imipenem |
|  |  |  | Penicillins: Ticarcillin, Témocillin | Aminoglycosides: Amikacin,  Gentamicin |
|  |  |  | Cephalosporins: Cefoxitin, Cefotaxime, Ceftazidime, Cefepime | Polymyxins: Colistin |
|  |  |  | Monobactams: Aztreonam |  |
|  |  |  | Carbapenems: Ertapénem |  |
|  |  |  | Quinolones: Nalidixic acid, Ofloxacin |  |
|  |  |  | Sulfonamides: Trimethoprim/Sulphamethoxazole |  |
| 6 | 8 | MDR | Penicillins+β-lactamase Inhibitors:  Amoxicillin/clavulanic acid,  Ticarcillin/clavulanic acid | Monobactams: Aztreonam |
|  |  |  |  | Aminoglycosides: Amikacin,  Gentamicin |
|  |  |  | Penicillins: Ticarcillin, Témocillin | Polymyxins: Colistin |
|  |  |  | Cephalosporins: Cefoxitin, Cefotaxime, Ceftazidime, Cefepime |  |
|  |  |  | Carbapenems: Ertapénem, Imipenem |  |
|  |  |  | Quinolones: Nalidixic acid, Ofloxacin |  |
|  |  |  | Sulfonamides:  Trimethoprim/Sulphamethoxazole |  |
| 1 | 1 | MDR | Penicillins+β-lactamase Inhibitors:  Amoxicillin/clavulanic acid,  Ticarcillin/clavulanic acid | Monobactams: Aztreonam |
|  |  |  | Penicillins: Ticarcillin, Témocillin | Aminoglycosides: Amikacin,  Gentamicin |
|  |  |  | Cephalosporins: Cefoxitin, Cefotaxime, Ceftazidime, Cefepime | Quinolones: Nalidixic acid, Ofloxacin |
|  |  |  | Carbapenems: Ertapénem, Imipenem | Polymyxins: Colistin |
|  |  |  | Sulfonamides:  Trimethoprim/Sulphamethoxazole |  |
| 1 | 1 | MDR | Penicillins+β-lactamase Inhibitors:  Amoxicillin/clavulanic acid,  Ticarcillin/clavulanic acid | Sulfonamides: Trimethoprim/Sulphamethoxazole |
|  |  |  | Penicillins: Ticarcillin, Témocillin | Polymyxins: Colistin |
|  |  |  | Cephalosporins: Cefoxitin, Cefotaxime, Ceftazidime, Cefepime | Aminoglycosides: Amikacin,  Gentamicin |
|  |  |  | Monobactams: Aztreonam |  |
|  |  |  | Carbapenems: Ertapénem, Imipenem |  |
|  |  |  | Quinolones: Nalidixic acid, Ofloxacin |  |
| 1 | 1 | MDR | Penicillins+β-lactamase Inhibitors:  Amoxicillin/clavulanic acid,  Ticarcillin/clavulanic acid | Carbapenems: Imipenem |
|  |  |  | Penicillins: Ticarcillin | Penicillins: Témocillin |
|  |  |  | Cephalosporins: Cefoxitin, Cefotaxime, Ceftazidime, Cefepime | Aminoglycosides: Amikacin,  Gentamicin |
|  |  |  | Monobactams: Aztreonam | Polymyxins: Colistin |
|  |  |  | Carbapenems: Ertapénem |  |
|  |  |  | Quinolones: Nalidixic acid, Ofloxacin |  |
|  |  |  | Sulfonamides:  Trimethoprim/Sulphamethoxazole |  |
