# Supplementary material for: Dissemination of carbapenemase-producing Enterobacterales in the community of Rawalpindi, Pakistan
Source: PLoS One. 2022 Jul 8;17(7):e0270707. doi: 10.1371/journal.pone.0270707 (PMC9269877; doi:10.1371/journal.pone.0270707)
Supplement: S4 Table — (DOCX) [file pone.0270707.s004.docx]

**S 4 Table: Correlation between the genomic content of antibiotic resistance genes (ARG) and observed phenotypic resistance**

| Antibiotics families | Phenotypically resistant isolates  n (%) | Isolates carrying acquired ARG  n (%) | Phenotypically susceptible isolates carrying ARG  n (%) |
| --- | --- | --- | --- |
| Carbapenems | 78 (100) | 73 (94) | 0 (0) |
| Monobactams | 62 (79) | 62 (79) | 0 (0) |
| Aminoglycosides | 24 (31) | 22 (28) | 1(1) |
| Sulfonamides/Trimethoprim | 73 (94) | 73 (94) | 2 (3) |
| Quinolones | 77 (99) | 17 (22) | 1 (1) |
| Polymyxins | 1 (1) | 1 (1) | 0 (0) |

n= number of isolates %= Percentage

For carbapenems, the ARG considered to confer resistance to carbapenems were *bla*_NDM-1,_ *bla*_NDM-5_, *bla*_NDM-7,_ *bla*_NDM,_(variant not specified), *bla*_OXA-181_ and *bla*_OXA-232._ For monobactams, the ARG were *bla*_CTX-M-15_, *bla*_CTX-M-139_, and different *bla*_CMY_ variants. For aminoglycosides (amikacin and gentamicin), aminoglycoside modifying enzymes (AME) – encoding genes such as *aac(3)-IIa* and 16S rRNA methylases such as a*rmA, rmtB1, and rmtF1* were detected. For sulfonamides/trimethoprim, the ARG were *sul* (dihydropteroate synthase) and *dfr* (dihydrofolate reductase). For quinolones, the ARG were *qnrS1, qepA4, qepA9, qnrB4*. For polymyxins, the gene was *mcr-1*.
