# Supplementary material for: Dissemination of carbapenemase-producing Enterobacterales in the community of Rawalpindi, Pakistan
Source: PLoS One. 2022 Jul 8;17(7):e0270707. doi: 10.1371/journal.pone.0270707 (PMC9269877; doi:10.1371/journal.pone.0270707)
Supplement: Attachment — Submitted filename: Response to Reviewers.docx [file pone.0270707.s005.docx]

RE: PLOS One

Title: Dissemination of Carbapenemase-producing Enterobacterales in the community of Rawalpindi, Pakistan

============================================================
Reviewer's comments:
Reviewer #1: I have some question/comments for the author to improve their manuscript.
1. Introduction: Situation of CPE/CRE in Pakistan should be mentioned.

*Response: In revised version of manuscript, the information regarding CPE situation in Pakistan has been added. Please see lines 73 – 75 at page No. 4.*

2. Line 110-118: How many isolates were selected? and what is the criteria to select?

*Response:* No. of isolates and selection criteria have been mentioned at page No. 5 (lines 113 -114).

3. Line 178-185: What is colistin susceptibility result? it is not mention in this part.

*Response:* *Colistin susceptibility results have been added on Page No. 9 (lines189-191).*

4. Line 230: % should be shown.

*Response:* *The percentages of IncX3 and IncFII incompatibility groups commonly associated with bla_NDM_* *(14) are mentioned in revised version on Page 11 (line 242).*

5. Discussion: Although this study showed only one case/isolate of mcr-harboring CPE but it is a signal to monitoring. The mcr-harboring CRE were documented in several literature. The author can discuss this detail to lead awareness of the clinician and infection control. Please see 'Paveenkittiporn et al., Whole-Genome Sequencing of Clinically Isolated Carbapenem-Resistant Enterobacterales Harboring mcr Genes in Thailand, 2016-2019. Front Microbiol. 2021 Jan 11;11:586368. doi: 10.3389/fmicb.2020.586368.". This might be useful to discuss because it report from the Asian country.

*Response:* *We have added details as suggested and thanks to the reviewer for suggesting this article. In the revised version, we have cited this paper in the discussion, to highlight the spread of mcr harboring CRE in Asian countries. (Page 15, lines 319-325).*

-----------------------------------------------------------------------------------------
Reviewer #2:

Reporting data on the detection of CPE in each country is necessary to understand the dissemination of CPE and to develop countermeasures. However, the description of the results is not clear in the text. It makes difficult to understand results and needs to be revised.
*Response:* *Your comments have been considered and revisions have been made throughout the text to have a clearer reading of the results.*

L. 186 - 208
The number of CPE is unclear. How many CPE strains were isolated, 73, or 78? Which genes were detected in the strains? Which species were detected? Authors should add a table summarizing the essential information from Fig. S3 to the text.
*Response: We have clearly mentioned the number of CPE in revised version. Page No. 9, lines 193-195. Fig. S3 has been converted to table (S1 Table) containing all essential information such as genes detected, species, sequence types and phylogroups.*

Methods, Patients and samples
Although the paper is about CPE in the Community, it includes strains detected after 72 hours of hospitalization. These should be excluded.
*Response:* *Yes, the paper mainly discusses the community carriage of CPE but we also followed up (where possible) for the acquisition of CPE during hospitalization. We consider that this data is also of value as it shows the genetic features of hospital associated isolates which were quite like community acquired isolates. Also, the reviewer # 3 has suggested to include the discussion of hospital acquired isolates, hence we are keeping this information in the revised version.*

L. 142 - 150
There is a problem with CRE quantification because the counts are made using cultured bacteria that have been previously subjected to ertapenem selection, which overestimates the number of CRE.
*Response: The samples have been cultured on ertapenem-supplemented media and have not been exposed to ertapenem prior to spreading onto the plate.*

Also, CRE quantification is not used for discussion and I do not understand the significance of its inclusion. It would be better to delete the descriptions and figures related to CRE quantification.

*CRE quantification has been included in Discussion. Page 15-16, L:333-337*

L. 215
Strains named Enterobacter cloacae complex should be re-identified using ANI and the correct species name filled in. Also, do not abbreviate the name of a genus that appears for the first time.
*Response:* *ANI of the three Enterobacter strains was calculated with FastAni (Jain, C., Rodriguez-R, L.M., Phillippy, A.M., Konstantinidis, K.T. & Aluru, S. Nat Commun 9, 5114 (2018), and following were the results:*

*ANI of 99.9369 for 240Ea with Enterobacter cloacae,
ANI of 99.9451 for 255A with Enterobacter cloacae
ANI of 99.934 for 259B with Enterobacter cloacae.*

*Hence the three strains are E. cloacae and changes have been made accordingly in the revised manuscript. (Page No. 3, line 71), (Page No. 9, line 199) and (Page No. 11, line 224).*

Fig.1
In the process of processing the 262 negative patients, 4 patients (negative:262-refused:98-not-hospitalized:63-ICU:3-sampled:94) are missing. Please check.
*Response:* *These 4 patients were found carbapenem resistant on ertapenem supplemented agar but were not carrying any carbapenemase gene (CPE negative 262: refused:98-not hospitalized: 63-ICU:3-sampled:94,while remaining 4 were CRE not CPE, hence missing). We have made changes in Fig. 1 to clear this confusion.*

Fig.3
It should be carefully explained where the "clade" is. Especially for the ST648 clade, it is difficult to understand where the clade is located on the tree. It would be better to add some outgroups and make root for easier reading.
*Response:* *Changes have been made as suggested in Fig. 3.*

In the stacked bar graph of Fig. S1, the number of samples and the results are stacked on top of each other, and the number may be twice as large. It needs to be redesigned.
*Response:* *Fig. S1 has been redesigned. Number of samples and results have been separated.*

In Fig. S2, S4, and S5, only the number of detections is listed, and information such as from which ST type of which bacteria species is not available. Please add this information.

*Response:* *All the information of the isolates (ST types, bacterial species) has been provided in detail in Table S1,while the ST types and species have also been updated in Figures S2, S3 and S4 as per reviewer’s suggestion.*
--------------------------------------------------------------------------------------------
Reviewer #3:

In this study the authors investigated the prevalence of carbapenemase-producing Enterobacterales (CPE) in patients attending a hospital from the community in Rawalpindi, Pakistan. Rectal swabs obtained from patients attending traumatology and gynaecology services without any preceding antibiotic exposure, hospitalisation or travel history in the last 3 months were plated on screening agar. Enterobacterales were identified by MALDI-TOF MS and antibiotic susceptibility testing and whole-genome sequencing performed.
The paper is well written and all the results are clearly presented. It presents valuable information on the prevalence of community CPE in Pakistan. WGS highlights the dominant sequence types carrying CPE genes and provides epidemiological information on plasmids carrying carbapenemase genes.
I have the following comments and suggestions for the authors:

METHODS:
Page 5, line 98: Antimicrobial susceptibility testing. It would add to the manuscript if the authors could perform susceptibility testing against newer agents, like aztreonam+avibactam and cefiderocol.
*Response: the association aztreonam+avibactam has been tested for a subset of CPE (NDM-5 producing E. coli), the results being reported in this article: "International circulation of aztreonam/avibactam-resistant NDM-5-producing Escherichia coli isolates: successful epidemic clones." Journal of global antimicrobial resistance 27 (2021): 326-328. Indeed, a significant number of strains combining NDM-5, CMY-42 and specific insertions motives in the PBP3 showed a decreased susceptibility to aztreonam-avibactam.*

*As for cefidérocol, it is not available in Pakistan. In France, we currently have a shortage for reagents required to perform microdilution tests (E-tests strips and discs being not recommended for céfidérocol testing). Nonetheless, we keep this in mind for our future work.*

RESULTS:
Sampling: Page 8, line 160-162: In this study a young patient cohort was investigated (average age 38 and 44 years). How does this compare to other studies from the region? Could this influence the finding?
*Response: We have added a paragraph regarding influence of young patient cohort on the findings in discussion part, also reference of few studies showing children and older people are at high risk of acquisition. We agree that the prevalence of CPE could be higher in at-risk populations such as elderly and young children. Page no. 15, L: 326-332.*

Prevalence of CPE carriage: Page 8, line 169: It would be interesting to add some information on whether the high community prevalence was reflected in disease burden in the hospital. How many of the patients required treatment for CPE? Were any of the hospital acquired cases considered to be linked?

*Response: Information has been added regarding situation of patients from which samples were collected on Page no. 9 (lines 181-182). Patients were admitted for or pregnancy-related planned consultation. We did not record the occurrence of infections during the follow-up, but given our results, such study would be interesting to implement. As for the connection between community and hospital-acquired strains, we only screened for hospital-acquired CPE in patients found negative at admission. To see whether community-acquired strains can cause hospital-onset infections, we should consider CPE-positive patients at admission. Of note, few community and hospital acquired cases were closely linked as mentioned on Page no. 11, lines 233-235.*

Phylogeny: Page 10, line 220: The authors comment on the maximal genetic distances but do not discuss genetic similarities. On the tree some of the sequences look like they are related? Did they find possible relatedness between community and hospital acquired strains indicating possible cross transmission? Could the authors comment on this?
*Response: This has been mentioned in the results section, Phylogeny at Page 11 (lines 234 – 237).*

Relative abundance: Page 11, line 239: The authors present data on quantification of CRE but do not discuss the finding. Did they expect a difference in the relative abundance between community-acquired CRE and hospital-associated CRE? Does this depend on whether the patient receives antibiotics? I suggest to briefly mention this in the discussion.

*Response: We have discussed these points in the discussion section on page No. 15-16 (lines 333-337).*

DISCUSSION:
Line 248: ‘CPE carriage is mostly associated with hospital environment’ Did the other studies undertaken in Pakistan focus on hospital acquired cases? It would be interesting to discuss isolates that were acquired in hospital here. On the tree they look spread out over the clades but closely related to some of the community isolates? What is known about hospital acquisition of CPE in hospitals in Pakistan? How many of the CPE positive patients did require CPE treatment? What impact does the high community carriage rate have on hospitals?
*Response: Limited data is available regarding community carriage of CPE in Pakistan and mostly studies are based on hospital isolates. Hospital acquired isolates have been mentioned in discussion, along with their relatedness with community associated isolates. Factors which are responsible for CPE acquisition in hospitals and impact high community carriage on hospitals have also been included in revised version. (please see Page No. 12-13, lines 260-269). Data about CPE positive patients requiring CPE treatment was not collected. The impact of high community carriage has been discussed on pages 12-13 (lines 260 – 269).*

Line 278: ‘Combination of resistance genes, virulence, and ability to easily disseminate…’ The authors mention virulence here but do not discuss virulence data for the observed ST types. How frequent is ST 167 found in clinical samples?
*Response: The word virulence has been removed from the description of ST 167. The prevalence and importance of ST 167 in clinical settings has been discussed in detail at page 14 (lines 298 – 300).*

Line 295/296: It would add to the value of the manuscript to be able to discuss cefiderocol susceptibilities here. It might not be available as an agent in Pakistan yet but with the high prevalence of metallo-b-lactamases it would be helpful to assess its activity against the isolates of the study.

*Response: Following the Reviewer 2’s comment on antibiotics potentially active on CPE, cefiderocol is not available in Pakistan as a drug or for testing. In France, we currently have a shortage for reagents required to perform microdilution tests (E-tests strips and discs being not recommended for céfidérocol testing). Nonetheless, we keep this in mind for our future work.*
*Besides, the association aztreonam+avibactam has been tested for a subset of CPE (NDM-5 producing E. coli), the results being reported in this article: "International circulation of aztreonam/avibactam-resistant NDM-5-producing Escherichia coli isolates: successful epidemic clones." Journal of global antimicrobial resistance 27 (2021): 326-328. Indeed, a significant number of strains combining NDM-5, CMY-42 and specific insertions motives in the PBP3 showed a decreased susceptibility to aztreonam-avibactam.*

============================================================
