# Supplementary material for: Dissemination of carbapenemase-producing Enterobacterales in the community of Rawalpindi, Pakistan
Source: PLoS One. 2022 Jul 8;17(7):e0270707. doi: 10.1371/journal.pone.0270707 (PMC9269877; doi:10.1371/journal.pone.0270707)
Supplement: Attachment — Submitted filename: Response to Reviewers.docx [file pone.0270707.s006.docx]

RE: PLOS One

Title: Dissemination of Carbapenemase-producing Enterobacterales in the community of Rawalpindi, Pakistan

============================================================
Reviewer's comments:

Reviewer #1: There is incorrect on lines 327-328 "A study from Thailand also reported similar findings of mcr-1 detection from asymptomatic human feces in 2012 [52]." That study is conducted on patients with variety specimens, no feces. The author should be check and carefully mentioned. Please revise this sentence again.

*Response: Thank you for pointing out this mistake. Correction has been made. Page 15, Line, 329.*

-----------------------------------------------------------------------------------------
Reviewer #2:

Overall, it has improved, but there was one point that was not answered satisfactorily because the previous my review was unclear.

L. 150-158 Methods, Quantification of CRE
The counting method regarding the total amount of Gram-negative bacteria is unclear. According to the description in the text, rectal specimens were first inoculated onto ertapenem-supplemented agar plates. Therefore, even if the specimens were cultured on agar plates without ertapenem, the total amount of Gram-negative bacteria may not have been counted accurately, and those selected for ertapenem may have been counted. In such a case, the number of Gram-negative bacteria would be low and the CRE would be overestimated, which raises questions about the reliability of the CRE-RA and is unacceptable. The method of counting total Gram-negative bacteria should be clearly explained.

*Response:* *Thank you for pointing out the confusion in methods. The rectal swabs were not directly plated on the plates. Instead they were thoroughly mixed in normal saline first and then 100 ul of the swab saline mix was plated on MacConkey agar with and without antibiotic directly and serial dilutions were also made to calculate relative abundance. This methodology was followed based on previously published methods in following articles: ‘Relative Fecal Abundance of Extended-Spectrum-β-Lactamase-Producing Escherichia coli Strains and Their Occurrence in Urinary Tract Infections in Women. Antimicrob. Agents Chemother.* **57**, 4512–4517 (2013)***’*** *and* ***‘****High Rate of Acquisition but Short Duration of Carriage of Multidrug-Resistant Enterobacteriaceae After Travel to the Tropics.*  *Clin. Infect. Dis.* **61**, 593–600 (2015)*’*

*The methods part has been re-phrased to make it more clear. Please see page No. 5, line 98 and page No. 7, lines 151-153.*
============================================================
