# Supplementary material for: Dissemination of carbapenemase-producing Enterobacterales in the community of Rawalpindi, Pakistan
Source: PLoS One. 2022 Jul 8;17(7):e0270707. doi: 10.1371/journal.pone.0270707 (PMC9269877; doi:10.1371/journal.pone.0270707)
Supplement: Attachment — Submitted filename: Response to Reviewer.docx [file pone.0270707.s007.docx]

**RE: PLOS One (PONE-D-21-36109R3)**

Title: Dissemination of Carbapenemase-producing Enterobacterales in the community of Rawalpindi, Pakistan

============================================================

Reviewer #4: Comments to authors:
The current study is interesting; however, the authors should address the following comments to improve the quality of the manuscript:

Please write the scientific names of bacterial pathogens and genes in the correct form all over the manuscript and the references section.

Response: Bacterial pathogens and genes names have been re-checked and corrected where needed, Page No. 2, lines 43-44 and Page No. 21-27, lines 459, 462, 466, 472, 521, 529, 540, 583, 626, 630, 633, 643,646, 650, 653, 660, 664, 668, 672, 679, 683, 688.

Title:
I think the work would benefit from the title that contains the main conclusion of the study (should be derived from the conclusion). Please modify the title.

Response: This manuscript has been reviewed two times previously and we already made changes based on previous reviews. Furthermore, we found dissemination of many clones of bacteria, not just one specific; hence we are keeping a generalized title.

Abstract:
The abstract must illustrate the used methods and the most prevalent results (give more hints about methods and results). Besides, rephrase the aim of the work and the main conclusion of your findings.

Response: Methods have been added, Page No. 2, lines: 38-42 while most prevalent results have already been described in abstract. Please see Page No. 2, lines 43-49 and this was reviewed two times previously and we had changed it based on previous reviews. The main conclusion is re-phrased; Pages No. 2-3, lines: 49-53.

Introduction: (it needs to be more informative):
Give a hint about the virulence factors, different infections caused by E. coli and K. pneumoniae, and the mechanism of disease occurrence.

Response: Virulence factors, infections**,** and mechanism of disease occurrence have been added in introduction to make it more informative. Please see Page No. 3, lines 57-64.
The authors should illustrate the public health importance concerning the emergence of multidrug-resistant (MDR) bacterial pathogens that reflect the necessity of new potent and safe antimicrobial agents. Several studies proved the widespread MDR- bacterial pathogens;
Authors could add the following paragraph:
Multidrug resistance has been increased all over the world that is considered a public health threat. Several recent investigations reported the emergence of multidrug-resistant bacterial pathogens from different origins including humans, birds, cattle, and fish that increase the necessity of new potent and safe antimicrobial agents. Besides, the routine application of the antimicrobial susceptibility testing to detect the antibiotic of choice as well as the screening of the emerging MDR strains. You are advised to cite the following valuable studies:

1. PMID: 33177849
2. PMID: 33188216
3. PMID: 30150182
4. PMID: 33947875
5. PMID: 32994450
6. PMID: 32497922
7. PMID: 33061472
8. PMID: 34445951
9. <https://doi.org/10.5114/ceji.2013.3774020> .
10. <https://doi.org/10.1016/j.aquaculture.2021.737643>
11 <https://doi.org/10.1016/j.foodcont.2021.108066>

Response: The suggested paragraph is a generalized information on AMR which is suitable for a study focusing on AMR and One health. Our manuscript is focused work investigating the carriage of CREs and CPEs in community and the related information on Enterbacterales and carbapenem resistance mechanisms have been described in the introduction. We have added information on virulence, and diseases as suggested in prior comments. We appreciated the advice to add valuable studies but the references advised above are not significantly relevant to our work and do not add any value to the work, hence these have not been added.
Rephrase the aim of the work to be clear and better sound.

Response: Aim of the work has been rephrased, Page No.4, lines 88-92.

# Material and methods: Add this subtitle: Bacterial Isolation and identification

# Response: Subtitle and relevant detail has been added. Page No. 5, line no. 104.

# Discuss in detail the methods of isolation and identification of Enterobacterales, especially, E. coli and K. pneumoniae. Besides, specific references should be added.

# Response: The methods of isolation have already been discussed, Page No.5, line no. 112-115 while identification methods of Enterobacterales with references have been added, Page No. 6 line no. 115-126.

# Add the company, city, and country of the used bacterial media and reagents that were used in the biochemical identification of isolates. Also, enumerate all used biochemical reactions.

# Response: Biochemical reactions, company, city, and country of all used bacterial media and reagents have been added; Page No. 6, line no. 118-125. Modify the subtitle to be: Antimicrobial susceptibility testing:

# Illustrate the antimicrobial classes of the tested antibiotics

# Response: Subtitle named antimicrobial susceptibility testing has been added, Page No. 6, line no. 128, and antimicrobial classes have been added to the material and method section under the heading of antimicrobial susceptibility testing; pages 6-7, line no. 130-141.

# The authors are advised to classify the tested isolates to MDR, XDR, and PDR as described by Magiorakos et al. Magiorakos AP, Srinivasan A, Carey RB, Carmeli Y, Falagas ME, Giske CG, et al. Multidrug-resistant, extensively drug-resistant and pandrug-resistant bacteria: An international expert proposal for interim standard definitions for acquired resistance. Clin Microbiol Infect. 2012; 18:268–81. doi:10.1111/j.1469-0691.2011.03570. x.

# Response: Response: Categories of isolates have been made to XDR, and MDR as described by Magiorakos et al., Page No.12, lines 247-250. No isolate was found to be PDR.

# The correlation between phenotypic and genotypic multidrug resistance should be performed

# Response: The work aimed to assess the carriage of CREs and CPEs in community. Antibiotic susceptibility were analyzed for all isolates (n=170) while the detailed genomic analysis was done for 78 isolates after de-duplication based on antibiotic susceptibility profiles. The assessment of methodologies in detecting the resistance was not the scope of the work hence the correlation of phenotypic and genotypic methods has not been carried out in this work.

# Add the following subtitle to the methods section and discuss the method in detail (Support the methods with specific references): Multilocus sequence typing (MLST), Statistical analyses: Add more details about the software used in the statistical analyses.

# Response: Subtitles and relevant detail of MLST with references (Page No. 7, lines 155-158) and Statistical analyses along with references, and detail of software has been added; Page No. 9 line no. 192-197. Results: Add this subtitle: Phenotypic characteristics of the recovered isolates: Illustrate in detail the phenotypic characteristics of the recovered E. coli and K. pneumoniae isolates.

# Response: Subtitle and characteristics of recovered Enterobacterales isolates have been added, Page No. 11, line no. 221-224. .

# Illustrate in a table the results of The Antimicrobial susceptibility testing

# Table of antimicrobial susceptibility testing results has been added in supporting material as S1 Table (Page No. 12, line No. 243 and Page No. 28, line 690.

# Illustrate in a new table the occurrence of MDR (Multidrug resistance) among the recovered isolates as the following (illustrate the names of the antimicrobial classes and different antibiotics): No. of strains%, Type of resistance R, MDR, and XDR Phenotypic multidrug resistance (Antimicrobial classes and different antibiotics). The antibiotic -resistance genes

# Response: Table showing Isolates categorization into XDR and MDR has been added in the supporting material (S2 Table: Page No. 28, line 691), showing number of strains, %, type of resistance and phenotypic multidrug resistance and susceptibility (Antimicrobial classes and different antibiotics), while detail of all antibiotic resistance genes has already been described in (S3 Table: Page No. 28, line no. 692).

# The correlation (Correlation coefficient) between phenotypic and genotypic multidrug resistance should be performed.

# Response: The work aimed to assess the carriage of CREs and CPEs in community. Antibiotic susceptibility were analyzed for all isolates (n=170) while the detailed genomic analysis was done for 78 isolates after de-duplication based on antibiotic susceptibility profiles. The assessment of methodologies in detecting the resistance was not the scope of the work hence the correlation of phenotypic and genotypic methods has not been carried out in this work. Add the supplementary Figures to the main manuscript.

# Response: All Supplementary figures have been added to main manuscript, Page No. 10, lines 215-217, Page No. 13, lines. 278-283, Page No. 15, lines.318-319, Page No. 16, line no. 326-328.

# Increase the resolution of all Figures (it should be 600 dpi).

# Response: Resolution of all figures has been increased to 600 dpi.

# Discussion: The authors are advised to illustrate the real impact of their findings without repetition of results.

# Response: Impact of study findings has been added to discussion, Page No.20, Line no. 413-417. Illustrate the different mechanisms of antimicrobial resistance in E. coli and K. pneumoniae.

# Response: Prevalent resistance mechanisms in CRE have already been discussed in Introduction, Pages 3-4, line no. 71-80.

# Conclusion Should be rephrased to be concise and sounded. A real conclusion should focus on the question or claim you articulated in your study, which resolution has been the main objective of your paper?

# Response: Conclusion has been improved according to suggestion, Pages 20-21, lines 427-435.
