# Supplementary material for: Dissemination of carbapenemase-producing Enterobacterales in the community of Rawalpindi, Pakistan
Source: PLoS One. 2022 Jul 8;17(7):e0270707. doi: 10.1371/journal.pone.0270707 (PMC9269877; doi:10.1371/journal.pone.0270707)
Supplement: Attachment — Submitted filename: Response to Reviewers.docx [file pone.0270707.s008.docx]

**RE: PLOS One (PONE-D-21-36109R3)**

Title: Dissemination of Carbapenemase-producing Enterobacterales in the community of Rawalpindi, Pakistan
============================================================

**Review Comments to the Author

Reviewer #1:** This revised manuscript has been addressing all points of the reviewer's comments. I satisfy the response of the authors.

------------------------------------------------------------------------------------------------------------------------------------

**Reviewer #2:** My concerns previously noted have been corrected, and together with the corrections indicated by Reviewer 4, the manuscript is much better. I have commented on the parts of the text that the author refused in response to Reviewer 4's remarks as follows.

The comment refused by authors: Title
Comments: No modification is required. I do not believe that the title change is necessary because, as the authors claim, the study investigated the genus Enterobacterales bacteria.

The comment refused by authors: References assigned by Reviewer 4
Comments: No modification is required. The references assigned by Reviewer 4 are biased in authorship and are not impartial. I believe they should NOT be added.

The comment refused by authors: Discuss in detail the methods of isolation and identification of Enterobacterales, especially, E. coli and K. pneumoniae.
Comments: Modification is required. Selection plates and concentration of antimicrobials used will affect the detection rate. It is a good idea to add this information to the “limitation” in discussion.

*Response: The methods of isolation and identification of Enterobacterales have already been discussed page No. 5-6, lines 102-125. The limitation of selection plates and concentration of antibiotics has been added to the limitation of study on Pages 19-20. lines 415-418.*

The comment refused by authors: The correlation between phenotypic and genotypic multidrug resistance should be performed
Comments: Modification is required. I think, Reviewer 4 may not have pointed out the methodologies. The paper does include antimicrobial susceptibility tests and a genetic study, which suggests that the necessary experiments have been performed. However, it does not present data linking the phenotypic antimicrobial susceptibility to the genotype. In particular, a number of strains have been found to be susceptible to monobactam, aminoglycosides, and imipenem, and the correlation with genotype needs to be discussed.

*Response: The data of the observed phenotypic resistance along with the genomic content of the antibiotic resistance genes has been added. Since this information is not in the scope of main findings of the study, this has been included in the supplementary information as S4 Table.*

The comment refused by authors: Illustrate the different mechanisms of antimicrobial resistance in E. coli and K. pneumoniae.
Comments: No modification is required. As the author claims, the drug resistance mechanism is introduced in the introduction, so there is no need to add it to the discussion again.

----------------------------------------------------------------------------------------------------------------------------------

**Reviewer #3:** (No Response)
